# Clinically probable paraneoplastic vasculitic peripheral neuropathy in primary central nervous system lymphoma: a case report

**DOI:** 10.1186/s12883-025-04285-3

**Published:** 2025-06-24

**Authors:** Zhuangzhuang Zhang, Chen Ling, Yingshuang Zhang, Weiping Sun, Zhaoxia Wang, Lingchao Meng

**Affiliations:** 1 Department of Neurology, Peking University First Hospital, Beijing, China; 2 Department of Neurology, Peking University Third Hospital, Beijing, China

**Keywords:** Primary central nervous system lymphoma, Paraneoplastic syndrome, Paraneoplastic vasculitic neuropathy, Case report

## Abstract

Primary central nervous system lymphoma (PCNSL) is a malignant tumor known to cause direct mass effects and various paraneoplastic syndromes. However, paraneoplastic vasculitic peripheral neuropathy is rarely associated with PCNSL. In this case report, we describe a patient with PCNSL who presented with probable paraneoplastic vasculitic peripheral neuropathy. The patient initially experienced recurrent pain and weakness in bilateral lower limbs for two years, followed by a sudden decline in consciousness over two days. A biopsy of the right sural nerve revealed probable vasculitic peripheral neuropathy, and the diagnosis of PCNSL was confirmed through brain biopsy.

**Supplementary Information:**

The online version contains supplementary material available at 10.1186/s12883-025-04285-3.

## Introduction

Vasculitis encompasses a diverse group of diseases characterized by perivascular inflammatory infiltration, leading to ischemic damage in affected tissues [1]. Vasculitic peripheral neuropathy is broadly classified into two categories: systemic vasculitic peripheral neuropathy and non-systemic vasculitic peripheral neuropathy. It typically presents as a recurrent, multifocal, axonal peripheral neuropathy and is primarily diagnosed through nerve biopsy [1]. Paraneoplastic vasculitic peripheral neuropathy is a rare paraneoplastic syndrome that manifests as symmetrical or asymmetrical sensory-motor axonal peripheral neuropathy. It is most commonly associated with small cell lung cancer and lymphoma, and its onset can precede, follow, or occur simultaneously with the diagnosis of the malignancy [2, 3].

Primary central nervous system lymphoma (PCNSL) is predominantly a diffuse large B-cell lymphoma (DLBCL) confined to the central nervous system (CNS), affecting the brain, spinal cord, cranial nerves, eyes and meninges [4]. It is a rare type of non-Hodgkin lymphoma, accounting for 1% of non-Hodgkin lymphomas and 3–4% of all brain tumors [5]. The clinical manifestation of PCNSL is diverse, including behavioral changes, memory and language impairments, seizures, increased intracranial pressure, and neuropsychiatric symptoms [6]. Beyond its space-occupying effects, PCNSL can also lead to a range of paraneoplastic syndromes, including dysautonomia [7], choreoathetosis [8], and myasthenia gravis [9]. However, paraneoplastic vasculitic peripheral neuropathy is rarely associated with PCNSL.

In this study, we presented a case of PCNSL accompanied by probable paraneoplastic vasculitic peripheral neuropathy.

## Case presentation

A 49-year-old man was admitted to the hospital with a two-year history of recurrent pain and weakness in bilateral lower limbs. Two years ago, he initially experienced subacute onset of right leg pain and weakness, which gradually progressed over the course of several weeks. Over the following one year, symptoms spread sequentially to the left lower limb, with persistent pain and weakness, while the upper limbs remained unaffected throughout the course of the disease. In addition to severe bilateral lower limb pain, he reported sensory disturbances, including decreased touch sensation and impaired deep sensation in both legs. The pain was neuropathic in nature—described as burning and tingling—and was localized to the bilateral lower extremities, without involvement of the back. At that time one year ago, which was one year prior to the current hospitalization, he visited a local hospital for medical attention. The physical examination revealed normal consciousness, higher cognitive functions, and cranial nerves. He exhibited hyperalgesia, along with reduced touch and deep sensation in both lower limbs. Muscle strength was graded 5/5 in bilateral upper limbs, 4/5 in the proximal lower limbs, and 3/5 in the distal lower limbs. The distribution of weakness was asymmetric and multifocal, predominantly affecting the distal muscles of the lower limbs in a non-dermatomal, patchy pattern, which is consistent with a mononeuritis multiplex-like presentation. Muscle atrophy was observed in bilateral lower limbs. Bilateral knee jerk reflexes and ankle jerk reflexes were absent, and pathological signs were negative bilaterally.

**Fig. 1:**
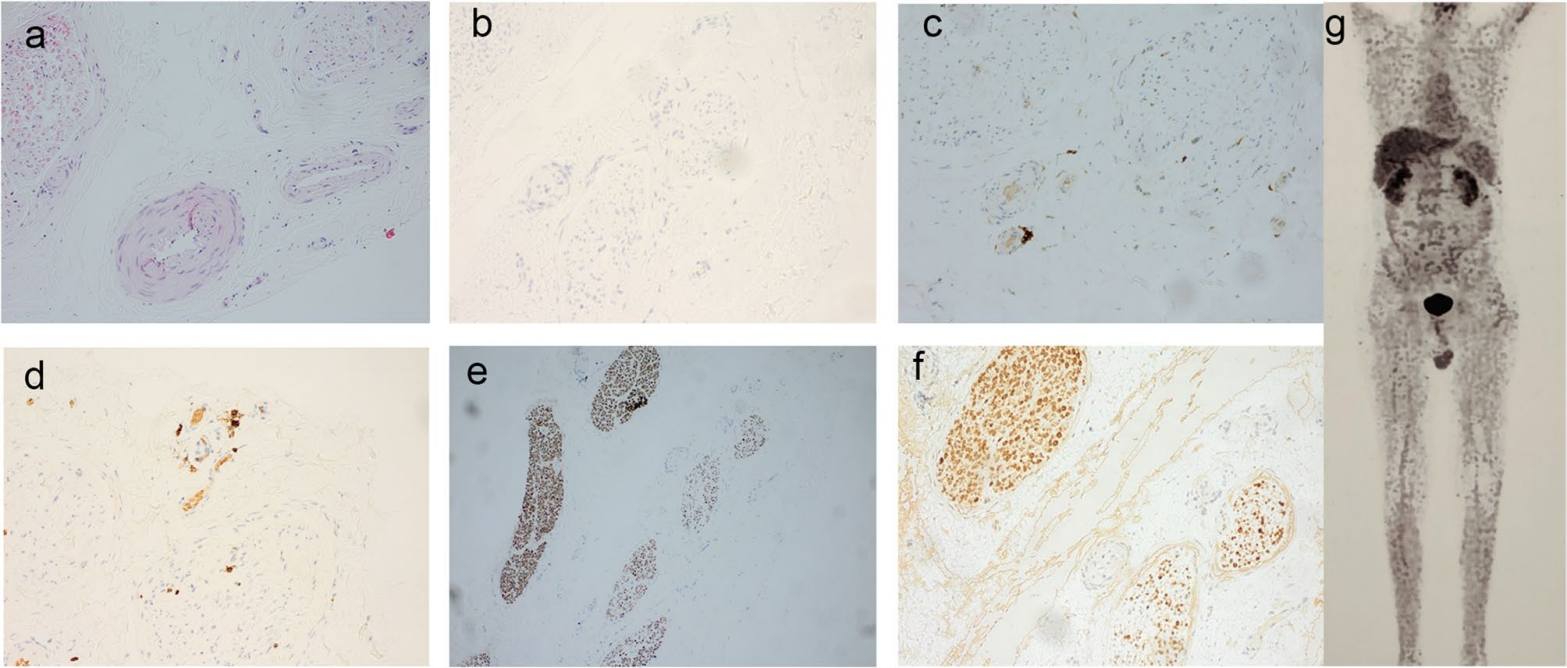
Right sural nerve biopsy and PET-CT (**a**) hematoxylin-eosin staining (200×) reveals perivascular inflammation. **b** CD20 staining (200×) shows no evidence of B lymphocyte infiltration around the vessels. **c** CD4 staining (200×) shows minimal perivascular infiltration by T lymphocytes. **d **CD8 staining (200×) also demonstrates minimal T lymphocyte infiltration around the vessels. **e** Neurofilament staining (100×) reveals marked variability in axonal degeneration across different nerve bundles. **f **Myelin basic protein staining (200×) reveals a reduction in large-diameter myelinated fibers. **g** No increased fluorodeoxyglucose uptake was observed in the peripheral nerves or nerve roots on PET-CT

At that time one year ago, which was one year prior to the current hospitalization, the nerve conduction study revealed reduced motor nerve conduction velocity, prolonged distal latency, and decreased compound muscle action potential (CMAP) amplitude in the bilateral tibial and common peroneal nerves of the lower limbs, while the motor conduction of the bilateral ulnar and median nerves of the upper limbs was normal. Similarly, sensory nerve conduction studies showed reduced conduction velocity, prolonged distal latency, and decreased sensory nerve action potential (SNAP) amplitude in the bilateral sural and superficial peroneal nerves of the lower limbs, with normal findings in the bilateral ulnar and median sensory nerves of the upper limbs. The results of the sensory and motor nerve conduction studies were asymmetric, with the right lower limb more severely affected than the left. The H-reflex of the right tibial nerve was lost. Needle electromyography examination revealed neurogenic damage in both the right tibialis anterior and left gastrocnemius muscles, manifested as prolonged duration, increased amplitude, and increased proportion of multiphase waves of motor unit action potentials when the muscles contracted slightly, and a significant decrease in the number of motor units when the muscles contracted forcefully. Laboratory tests revealed normal results for routine blood tests, liver and kidney function, coagulant function, high-sensitivity C-reactive protein, and erythrocyte sedimentation rate. Anti-nuclear antibody was positive with a speckled pattern at a titer of 1:80. Anti-ENA antibodies and anti-neutrophil cytoplasmic antibodies were negative. Serum and urine immunofixation electrophoresis were normal. Carcinoembryonic antigen was 6.1 ng/mL (normal range < 5 ng/ml), and tumor marker CA724 was 18.8 U/mL (normal range < 6.9 U/mL). Cerebrospinal fluid (CSF) opening pressure and nucleated cell count were within normal limits, with a CSF protein concentration of 0.9 g/L (normal range < 0.45 g/L). CSF oligoclonal bands and all anti-ganglioside antibodies were negative. The patient underwent a biopsy of the right sural nerve. The nerve biopsy revealed inflammatory cell infiltration around epineurial blood vessels, marked axonal degeneration, and a decreased density of myelinated nerve fibers within the nerve fascicles, with variability observed between different fascicles (Fig. 1). Although classic features of vasculitis such as transvascular inflammatory cell infiltration or vessel wall destruction were not definitively observed, the pathological findings, together with the clinical presentation were suggestive of a probable vasculitic peripheral neuropathy. The patient experienced partial symptom improvement with treatment consisting of glucocorticoids (65 mg daily), tacrolimus (1.5 mg twice daily), and cyclophosphamide (100 mg daily), but his condition worsened when the steroid dosage was tapered.

**Fig. 2:**
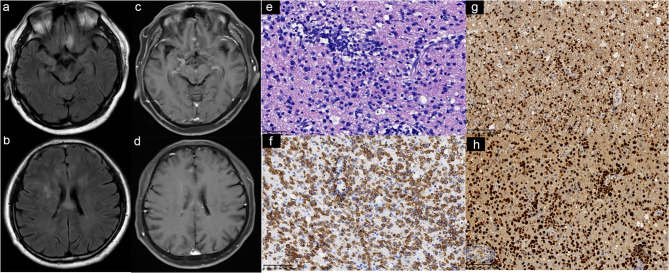
Brain MRI and left frontal lobe biopsy reveals primary central nervous system diffuse large B-cell lymphoma. Brain MRI revealed hyperintense signals on T2-FLAIR sequences (**a**, **b**) in the bilateral frontal lobes, corona radiata, basal ganglia, and corpus callosum, with partial areas showing punctate enhancement on T1-weighted contrast-enhanced imaging (**c**, **d**). **e** Hematoxylin and eosin (H&E) staining (400×) shows atypical lymphocytes that are relatively large, round or oval, and appear either individually scattered or in clusters. **f**-**g** The atypical lymphocytes are positive for CD20 (200×) (**f**) and PAX5 (200×) (**g**). **h** The Ki-67 labeling index is approximately 70% (200×)

Two days before the current admission, he experienced a sudden decline in consciousness, characterized by drowsiness and significantly slowed responses. His medical and family histories were unremarkable. At admission, he was conscious but apathetic, with sluggish reactions. Moreover, touch, pain, proprioception, kinaesthesia, and vibration perception were diminished in bilateral lower limbs. Muscle strength in the bilateral upper limbs was 5/5, while the proximal and distal muscle strength in the lower limbs were 4/5 and 2/5, respectively. Brain MRI revealed hyperintense signals on T2-FLAIR sequences in the bilateral frontal lobes, corona radiata, basal ganglia, and corpus callosum, with partial areas showing punctate enhancement on T1-weighted contrast-enhanced imaging (Fig. 2). Routine blood, urine, and stool tests revealed no abnormalities. His erythrocyte sedimentation rate was 26 mm/h (normal range 0–15 mm/h). Liver and kidney function tests, as well as creatine phosphokinase and its isoenzyme levels, were all within normal limits. Thyroid function, thyroid-related antibodies, tumor markers, cryoglobulins, immunoglobulins, complement levels, lymphocyte subpopulations, and immunofixation electrophoresis in blood and urine were also unremarkable. Antinuclear antibody was positive at a titer of 1:100 with a granular pattern, while tests for extractable nuclear antigen antibodies, antineutrophil cytoplasmic antibodies, rheumatoid factor, antistreptolysin O, and antiphospholipid antibodies were negative. Serum lactate dehydrogenase was elevated to 312 U/L (reference range: 100–240 IU/L).

The lumbar puncture results showed that the opening pressure, white blood cell count, and glucose levels in the CSF were within normal ranges. However, the CSF protein level was elevated at 0.86 g/L (normal range < 0.45 g/L), and tests for anti-cytomegalovirus and anti-Epstein-Barr virus antibodies were negative. CSF flow cytometry revealed an increase in leukocytes, predominantly lymphocytes, with all lymphocytes positive for CD20 and 57% positive for CD19. Autoimmune-encephalitis-related antibodies, anti-aquaporin 4-antibody, myelin oligodendrocyte glycoprotein antibody, and antiganglioside antibodies were all negative in both serum and CSF. Oligoclonal bands were positive in both CSF and serum. Further CSF analysis revealed the presence of anti-paraneoplastic antibodies, specifically anti-Hu antibody, increased blood–brain barrier permeability (10.98 × 10^−3^; normal range < 5), an elevated IgG index (0.89; normal range < 0.85), and an increased intrathecal IgG synthesis rate (14.52 mg/24 h; normal range < 7 mg/24 h). Other paraneoplastic autoantibody testing, including ampiphysin and collapsin-response-mediator-protein-5 antibodies, was performed and both were negative, helping to exclude other causes of asymmetric painful polyradiculoneuropathy.

Nerve conduction studies (Table 1) revealed absent motor responses in the bilateral tibial and common peroneal nerves of the lower limbs, while motor conduction in the bilateral ulnar and median nerves of the upper limbs remained normal. Sensory conduction studies showed absent responses in the bilateral sural and superficial peroneal nerves, whereas sensory conduction in the bilateral ulnar and median nerves was normal. Needle electromyography revealed neurogenic damage in the tibialis anterior, gastrocnemius, biceps femoris, and quadriceps muscles, characterized by prolonged duration, increased amplitude, and higher polyphasic wave proportions of the motor unit action potentials during mild muscle contraction. During forceful muscle contraction, there was a marked reduction in the number of motor units. Fibrillation potentials and positive sharp waves were observed in the right tibialis anterior and left gastrocnemius muscles. Needle electromyography was also performed on the biceps brachii and abductor digiti minimi of both upper limbs, showing no significant abnormalities and no evidence of myogenic or neurogenic damage. The electroencephalogram (EEG) showed a slow and irregular alpha wave pattern, with reduced prominence of the alpha wave in the left occipital region compared to the right, as well as a slower wave frequency. Positron emission tomography-computed tomography revealed multiple lesions with increased glucose metabolism in the bilateral frontal lobes, corona radiata, basal ganglia, and corpus callosum, but no such lesions were detected in the visceral organs, peripheral nerves, or nerve roots (Fig. 1). A biopsy of the left frontal lobe was performed (Fig. 2), with immunohistochemistry revealing CD20(+), PAX5(+), CD10(+), BCL-6(+), MUM1(+), CD3(-), CD56(-), BCL-2(-), CD68(-), GFAP(-), and Olig2(-). The Ki-67 antigen labeling index was approximately 70%, and the C-MYC labeling index was around 30%.

**Table 1:** Electrophysiological findings Pre- and Post-Treatment

	**Pre-treatment**			**Post-treatment**		
	**DML ** **(ms)**	**CMAP** **(mV)**	**MCV** **(m/s)**	**DML** **(ms)**	**CMAP** **(mV)**	**MCV** **(m/s)**
Moter Nerve						
Median L						
W-APB	3.4	12.9		3.28	8.68	
E-W	8.06	11.9	52.6	7.6	7.1	55.5
Median R						
W-APB	3.27	11.2		3.28	7.28	
E-W	7.58	10.3	52.2	7.5	7.17	56.9
Ulnar L						
W-ADM	2.54	8.3		2.76	6.73	
E-W	6.84	7.5	52.3	6.45	5.03	59.5
Ulnar R						
W-ADM	2.6	12		2.71	8.52	
E-W	6.33	11.4	56.3	6.42	6.22	57.4
Peroneal L						
A-EBD	NR			NR		
CF (Down 2cm)-TA	NR			4.9	0.62 ↓	
CF (UP 9cm)-CF (Down 2cm)	NR			6.51	0.91 ↓	43.4
Peroneal R						
A-EBD	NR			NR		
CF (Down 2cm)-TA	NR			NR		
Tibial L						
A-AH	NR			NR		
PF-A	NR			5.42	1.38 ↓	
Tibial R						
A-AH	NR			NR		
PF-A	NR			NR		
	**DML** **(ms)**	**SNAP** **(uV)**	**SCV** **(m/s)**	**DML** **(ms)**	**SNAP** **(uV)**	**SCV** **(m/s)**
Sensory Nerve						
Median L						
IIID-W	2.64	9.5	56.8	2.4	36.2	56
Median R						
IIID-W	2.85	12.7	56.1	2.7	18.6	54
Ulnar L						
VD-W	2.21	6.4	49.8	2.2	16	52.1
Ulnar R						
VD-W	2.61	9.3	44.1	2.27	19.6	49.3
Sural L						
MLL-LM	NR			NR		
Sural R						
MLL-LM	NR			NR		
Superficial peroneal L						
LLL-LM	NR			NR		
Superficial peroneal R						
LLL-LM	NR			NR		

*IIID* Third digit, *A* Ankle, *ADM* Abductor digiti minimi, *AH* Abductor hallucis, *APB *Abductor pollicis brevis, *CF* Capitulum fibula, *CMAP* Compound muscle action potential, *DML* Distal motor latency, *E* Elbow, *EBD* Extensor digitorum brevis, *L* Left, *LLL* Lateral lower leg, *LM* Lateral malleolus, *MCV* Motor nerve conduction velocity, *MLL* Middle lower leg, *NR* Not recordable, *PF* Popliteal fossa, *R* Right, *SCV* Sensory nerve conduction velocity, *SNAP* Sensory nerve action potential, *TA* tibialis anterior, *W* Wrist, *VD* Fifth digit

The patient was ultimately diagnosed with primary central nervous system-diffuse large B-cell lymphoma (PCNS-DLBCL). Multiple diagnostic tests, including chest CT, abdominal ultrasound, and superficial lymph node ultrasound, indicated no evidence of a tumor. As the patient’s vasculitis was confined to the peripheral nerves, with no evidence of multi-organ involvement, and in the absence of a history of diabetes or toxicity, other etiologies of vasculitic neuropathy, such as systemic vasculitis, toxicity, and diabetic neuropathy, were excluded. In the absence of direct tumor infiltration into the peripheral nerves, the probable vasculitic peripheral neuropathy was considered to be a paraneoplastic syndrome caused by PCNS-DLBCL. Following treatment with rituximab, methotrexate, and zanubrutinib, the patient’s memory, consciousness, and responsiveness improved. At the one-year follow-up, the patient’s lower limb pain had significantly improved. Muscle strength in the proximal lower limbs remained at grade 4, while distal lower limb strength improved from grade 2 to grade 3. Repeated nerve conduction studies (Table 1) showed that motor and sensory conduction in the bilateral median and ulnar nerves remained normal. Sensory conduction in the bilateral sural and superficial peroneal nerves of the lower limbs remained absent. However, motor nerve conduction in the left lower limb showed improvement compared to the previous study. The motor conduction of the left common peroneal nerve, recorded from the tibialis anterior, changed from an absent to a reduced CMAP amplitude. Similarly, the motor conduction of the left tibial nerve, recorded from the gastrocnemius muscle, showed a change in CMAP amplitude from absent to reduced.

## Discussion

In this case, we report a patient who presented with probable vasculitic peripheral neuropathy and was later diagnosed with PCNS-DLBCL. The differential diagnosis of vasculitic peripheral neuropathy, characterized by painful sensorimotor axonal neuropathy with an asymmetric pattern, is extensive, with paraneoplastic syndrome being a less common etiology. Paraneoplastic peripheral neuropathy refers to neuropathies that arise from the distant effects of a tumor, unrelated to direct tumor infiltration [10]. Previous studies have shown that lymphoma, including angioimmunoblastic T-cell lymphoma, can cause paraneoplastic peripheral neuropathy [11]. As a primary tumor, PCNS-DLBCL has been known to cause various paraneoplastic syndromes, such as opsoclonus-myoclonus syndrome [12]. However, there are few reports of vasculitic peripheral neuropathy as a paraneoplastic manifestation of PCNS-DLBCL, a rare form of primary extranodal non-Hodgkin lymphoma [13]. This case highlights the potential for PCNS-DLBCL to cause paraneoplastic vasculitic peripheral neuropathy and should be considered in cases of refractory vasculitic neuropathy. The primary treatment involves high-dose methotrexate-based chemotherapy, with high-dose chemotherapy followed by autologous stem cell transplantation showing effectiveness as consolidation therapy. Additionally, targeted therapies like imatinib and lenalidomide have demonstrated efficacy. Despite treatment, the prognosis remains poor, with high recurrence rates and increased mortality [14].

Although pathological features such as fiber density variability among fascicles and mild perivascular inflammation were observed, these findings are not specific for vasculitis and may also be seen in other disorders such as chronic inflammatory demyelinating polyneuropathy variants. Therefore, the possibility of paraneoplastic vasculitic neuropathy is suggested rather than definitively confirmed. Based on the diagnostic criteria for non-systemic vasculitic neuropathy [15], the patient was ultimately diagnosed with probable vasculitic neuropathy. Previous studies have shown that paraneoplastic neuropathy can occur at any stage of malignancy and should be considered in all patients with cancer [3]. Paraneoplastic peripheral neuropathy typically presents with neurological symptoms prior to the detection of malignancy [16, 17]. Therefore, comprehensive and early tumor screening is crucial [18]. In this case, the peripheral neuropathy preceded the identification of the malignancy, highlighting that even in the absence of initial tumor evidence, regular screening remains essential.

PCNSL rarely presents with systemic paraneoplastic syndromes. Most of the available literature focuses on cases with direct tumor infiltration or localized neurological manifestations. The types of peripheral neuropathies associated with PCNSL are highly diverse [11]. A previous case report has described demyelinating peripheral neuropathy related to PCNSL, potentially linked to direct tumor invasion and paraneoplastic immune processes [19]. In contrast, our patient presented with probable vasculitic peripheral neuropathy associated with PCNSL, likely related to paraneoplastic immune mechanisms. Further research is needed to expand the spectrum of peripheral neuropathies related to PCNSL and to elucidate the underlying mechanisms of peripheral nerve involvement. To date, there are limited case reports describing systemic vasculitic neuropathy as a paraneoplastic syndrome in PCNSL. The pathogenesis underlying this phenomenon remains unclear, but immune dysregulation triggered by PCNSL is thought to play a critical role. Specifically, tumor-induced activation of pro-inflammatory cytokines or autoantibody production may lead to vasculitic changes in peripheral nerves. Further research is warranted to elucidate the mechanisms involved.

In conclusion, we report a case of PCNSL associated with probable paraneoplastic vasculitic peripheral neuropathy. The patient’s symptoms improved following treatment with rituximab, methotrexate, and zanubrutinib. Although rare, paraneoplastic vasculitic neuropathy associated with PCNSL is a possibility that warrants further investigation.

## Supplementary Information

Supplementary Material 1.

## Data Availability

No datasets were generated or analysed during the current study.

## Abbreviations

IIID: Third digit
A: Ankle
ADM: Abductor digiti minimi
AH: Abductor hallucis
APB: Abductor pollicis brevis
CF: Capitulum fibula
CMAP: Compound muscle action potential
CNS: Central nervous system
CSF: Cerebrospinal fluid
DLCBL: Diffuse large B-cell lymphoma
DML: Distal motor latency
E: Elbow
EBD: Extensor digitorum brevis
EEG: Electroencephalogram
L: Left
LLL: Lateral lower leg
LM: Lateral malleolus
MCV: Motor nerve conduction velocity
MLL: Middle lower leg
NR: Not recordable
PCNSL: Primary central nervous system lymphoma
PF: Popliteal fossa
R: Right
SCV: Sensory nerve conduction velocity
SNAP: Sensory nerve action potential
TA: tibialis anterior
W: Wrist
VD: Fifth digit
